# Pressures received and generated by a group of workers with subsistence jobs in the center of Medellín, and how these affect their life and health

**DOI:** 10.47626/1679-4435-2022-970

**Published:** 2024-02-16

**Authors:** María Osley Garzón-Duque, Deisy Soledad Monsalve-Bedoya, Fabio León Rodríguez-Ospina, Doris Cardona-Arango, Ángela María Segura-Cardona, Sara Toro-Tobón

**Affiliations:** 1 Universidad CES, Medellin, Colombia, Facultad de Medicina, Medellin, ANT, Colombia; 2 Universidad de Antioquia, Facultad Nacional de Salud Pública, Medellin, ANT, Colombia; 3 Universidad CES, Medellin, Colombia, Escuela de Graduados, Facultad de Medicina, Medellin, ANT, Colombia; 4 Universidad CES, Medellin, Colombia, Escuela de Graduados, Medellin, ANT, Colombia

**Keywords:** informal sector, qualitative research, heath, disease, working environment, trabajadores informales, investigación cualitativa, salud, enfermedad, ambiente de trabajo

## Abstract

**Introduction:**

Although studies about informal work have been carried out, there is still little evidence that explains, from the workers' perspective, what pressures they receive and generate due to the use of public space, and how these pressures affect their health.

**Objectives:**

To explore, from the point of view of a group of informal workers from the downtown Medellin, the environmental and social pressures that they receive and generate from the use of the territory, as well as the effects that these pressures may have on their life and health conditions.

**Methods:**

Ethnographic tools were used for field work and grounded theory for data analysis. Twelve informal street vendors workers were selected through theoretical sampling, with whom in-depth interviews and focus groups were conducted, after obtaining consent from the verbal and written process. Interviews and focus groups were transcribed verbatim, with the help of janitors and informants. The results were discussed and validated with the workers, and the information was triangulated with the researchers. Open and axial coding was used for data analysis.

**Results:**

The environmental and social pressures that these workers receive and generate in the streets and sidewalks of the city led them to experience critical situations in their working conditions, partly derived from the conflict that occurs over the use of the territory with the different actors in the downtown area, a situation that directly affects workers' physical and mental conditions, their life, and their work.

**Conclusions:**

The conflicts generated by the use of the territory as a workplace imply that workers have hostile relationships in their daily lives. However, these conflicts could be resolved with actions of the State and the participation of workers.

## Abstract (es)

**Introduction:**

Aunque se han realizado estudios acerca del trabajo informal, aun es escasa la evidencia que explique, desde la mirada de los trabajadores, qué presiones reciben y generan por el uso del espacio público y cómo estas presiones les afectan su salud.

**Objetivos:**

Explorar, desde la mirada de un grupo de trabajadores informales del centro de Medellin, las presiones ambientales y sociales que reciben y generan por el uso del territorio, así como los efectos que pueden tener estas presiones en sus condiciones de vida y salud.

**Métodos:**

Se utilizaron herramientas etnográficas para el trabajo de campo y de teoria fundamentada para el análisis de datos. Se tomaron mediante muestro teórico a 12 trabajadores informales “venteros”, con quienes se realizaron entrevistas en profundidad y grupos focales, previa toma de consentimiento de proceso verbal y escrito. Se transcribieron las entrevistas y grupos focales de manera textual, se contó con porteros e informantes clave. Los resultados fueron discutidos y validados con los trabajadores, y se trianguló la información con los investigadores. Se utilizó codificación abierta y axial para el análisis de datos.

**Resultados:**

Las presiones ambientales y sociales que reciben y generan estos trabajadores en las calles y aceras de la ciudad los llevan a vivenciar situaciones críticas en sus condiciones laborales, derivadas, en parte, del conflicto que se da por el uso del territorio con los diferentes actores del centro de la ciudad, situación que afecta directamente las condiciones de salud física y mental de los trabajadores, su vida y su labor.

**Conclusiones:**

Los conflictos que se generan por el uso del territorio como lugar de trabajo implican que los trabajadores tengan relaciones hostiles en su cotidianidad. Sin embargo, estos conflictos podrían revertirse con acciones del Estado y la participación de los trabajadores.

## INTRODUCTION

In a research conducted in 2015, the Organisation for Economic Co-operation and Development reported that, for 2020, the informal sector would account for 66.0% of the labor force,^1^ and that working conditions affect workers' emotional, physical and mental state, with rates of diseases and injuries being consistent with workers' exposures,^1^ according to their occupation in the informal sector, which includes individuals who make streets and sidewalks into their workplace.

Informal employment is part of the social response to scarcity of formal jobs.^2^ In relation to regulatory framework and informal economy, the International Labor Organization mentions the importance of implementing changes in the government structure, with collective initiatives including the participation of vendors, street vendors in this case, in regulating and protecting their assets, contributing to the common good of society.^3^

In Colombia, the public space is often used as a workplace and thus also represents a means of subsistence that could lead to improved quality of life for workers and their families.^2^ However, their occupation of what is considered a public good generates a conflict between the use of the territory as a workplace and as a public good and the place for the development of several daily life activities.^2^ In the third trimester of 2020, according to the National Administrative Department of Statistics (Departamento Administrativo Nacional de Estadística, DANE), the percentage of informal workers was 47.2% for 23 municipalities in Colombia,^4^ a percentage slightly lower than that reported in Latin America and the Caribbean, where the percentage of informal workers was 53.0%, accounting for nearly 140 million workers.^5^

Use of public space as a workplace can lead to hostile relations. In Medellin, work activities in public space were considered for the first time in 1987 within the first Urban Development Plan,^6^ resulting in situations of insecurity and massive increase in the number of street vendors. This scenario gave rise, on one hand, to proposals for the need for the State to recover public space,^6^ and, on the other, to claims from street vendors in the downtown area that that public space was their workplace and the survival option for themselves and for those for whom they are responsible.^2^ The foregoing has also led to the creation of some guidelines for the use of urban public space, which established that, for the case of informal street vendors, a census should be conducted to obtain data related to family groups, households, and health, etc., required to investigate the possibility of granting permission to use public space as their workplace, in addition to the need for complying with criteria set forth in Decree 725 of 1999,^7^ which include compliance with the Antioquia's Police Code^7^ and the regulatory standards for vendors.^7^

Informal sales in the city of Medellin, Colombia, have been a recurrent theme of interest in public and occupational health, partly evidencing mobility difficulties in streets and footpaths, as well as the different and difficult actions/interactions experienced by workers, affecting their life and health conditions. For the abovementioned reasons, this study aimed to describe, from the perspective of a group of informal street vendors in downtown Medellin, Colombia, from 2015-2019, environmental and social received and generated by them due to use of public space as a workplace, and the effects that these pressures may have on their life and health.

## METHODS

### INTERPRETATIVE FRAMEWORK

The present study was addressed using a naturalistic approach, since it was focused on understanding workers' experiences and meanings with regard to pressures received and generated from the use of public space as a workplace, as well the effects that these pressures may have on workers' life conditions and health. To this end, qualitative ethnographic research tools were used for the field work; similarly, grounded theory was used for data analysis, and an action research approach was used during the entire process.

### POPULATION AND SAMPLING

The reference population was 686 informal street vendors in downtown Medellín, Colombia, who participated in the general study described in the doctoral thesis from which the present article derives. From this population, a group of participants was selected through snowball sampling, and then a theoretical sampling was conducted with 12 workers, based on information needs derived from the first stages of research. The study included workers capable of verbally expressing their experiences and meanings with regard to the themes of interest, older than 18 years, with more than 10 years of professional experience, who were informed about the study and its procedures, and who accepted to participate through verbal and written consent before data collection. No participant was lost according to the established criteria.

### DATA COLLECTION INSTRUMENTS

An interview script, a focus group script, and an observation guide were used, and a field diary was kept to record data collection activities. The instruments were designed and validated with the workers and their leaders from 2015 and 2017, in a process of collective knowledge construction. Eleven interviews and a focus group were conducted. For fieldwork, an immersion was carried out with workers and their leaders, with the support of janitors and key informants, with which authors have been working since 2007.

### THEMES OF ANALYSIS

Pressures received by workers in the public space as their workplace, pressures generated by workers in the public space, and effects of the received pressures on their life and labor, as well as actions promoted by the State, workers, their families, and leaders.

### RIGOR CRITERIA

A field immersion and approach with workers were carried out; moreover, constructed and validated instruments were used with participants, and a field diary was kept during observations. Interviews and the focus group were recorded and textually transcribed, and data analysis took into account the logics of ground theory. Furthermore, results were triangulated with researchers and participants, with whom information was also validated, upon presentation of results to academic and institutional authorities.

### DATA ANALYSIS

Data were analyzed by open and axial coding throughout the entire study period, from interview and initial focus group in 2015 and 2016, continuing in 2017 and 2018, up to text structuring in 2018. Discussion and result validation occurred from 2018 to 2019. Theoretical codes were used to establish connections between categories and subcategories in axial coding. Data were recorded in diagrams and maps, and finally results were reported in prose.

### ETHICAL CONSIDERATIONS

The present article is a subproduct of primary data from the doctoral thesis “Vulnerabilidad socio laboral y ambiental de un grupo de trabajadores informales ‘venteros’ del centro de Medellín, bajo el modelo de fuerzas motrices. Medellín 2015-2019”, approved by the institutional Research Ethics Committee of Universidad CES, through protocol No.84 of 2015. The present study was classified as posing minimum risk, according to Resolution 8430, and took into account international standards that include the vulnerable population established by the Council for International Organizations of Medical Sciences (CIOMS).

## RESULTS

### THEY PRESS, ARE PRESSED, AND ENTER IN CONFLICT WITH THE STATE FOR TERRITORY

Unemployment in Colombia, derived, among other aspects, from displacement and violence, has led to an increase in the number of informal workers in the streets, who use public space as a workplace, and that is where they exert and receive pressures, entering in conflict with the State and with other actors for the use of public space as a source of employment, in the struggle for their own survival and that of the persons for which they are responsible, in addition to undergoing relocation processes, in which; "with the completion of Plaza Minorista (Minorista Square) in the 1980s, we were allocated to some points of sale...” (HE2O), at places where their products did not sell, and in many cases vendors ended up losing their capital, which was one of the reasons for not accepted the assigned positions. They acknowledged that "it was a difficult step, because, after people had spent their entire life working in Guayaquil, moving to some place there... Many people returned, because they were bankrupt” (HE2O). Furthermore, street vendors did not have their rights guaranteed, which generated frustration and fear: "We had though experiences before we got a labor permit, with the ESMAD (Mobile Anti-Disturbance Squadron, Escuadrón Móvil Antidisturbios in Spanish)” (EMVA1).

**Figure 1:**
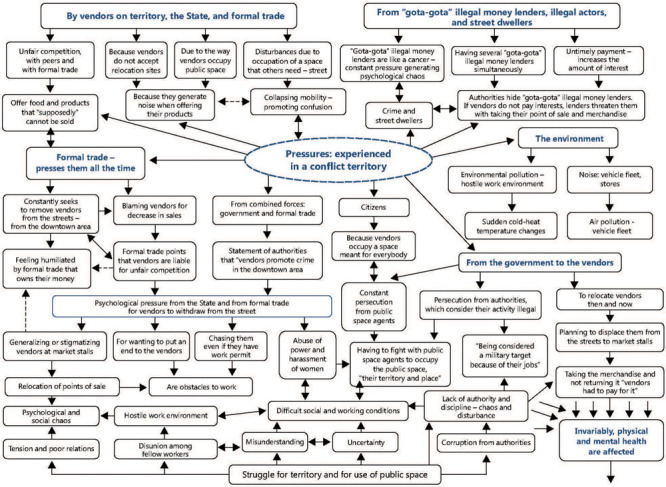
Pressures. Workers press, are pressed, and enter in conflict with different actors.

Furthermore, they feel they work amid anarchy, reporting that: “At this time, there is constant anarchy here, we see that the police, or the institution, was not in command, and this worries us very much, we don't know to whom we should talk.” (HGFG) (Figure 1).

### FORMAL TRADE AND PEDESTRIANS

Workers coexist with pedestrians and fellow workers in the downtown area and acknowledge that conditions are not the ideal ones; however, they must work by day to eat at night, thus having a subsistence job, and acknowledging also that they occupy a space and disturb other people. “Within public space, I'm an obstacle to formal vendors and to many other people who tell us that our presence in the streets are disturbing pedestrians, formal (workers), and other people.” (MGFLI) (Figure 1).

### ILLEGAL ACTORS (“GOTA-GOTA” ILLEGAL MONEY LENDERS AND DRUG DEALERS), THEIR PARTNERS, STREET DWELLERS, AND SEX WORKERS

Vendors also acknowledge that they must share public space with those who provide illegal loans with interests: “gota-gota” illegal money lenders or loan sharks; “we also have “gota-gota” illegal money lenders or loan sharks, for us they are a freaking constant pressure, it’s a psychological chaos, because if we don’t sell, we’re not able to pay, we have a double problem here” (HGFG).

Vendors press and are pressed by social environment; furthermore, because of the conditions under which they work, they are more vulnerable to the pressures received, a situation that has led to changes, coexistence difficulties, and hostile relations in their work environment. Workers themselves acknowledge: “...we are collapsing mobility in the downtown area and unintentionally promoting disturbance in the municipality, because together with vendors also there are also streets dwellers, criminals, and prostitutes, that is, we are unintentionally pressing for the creation of chaos in the society” (HE1A) (Figure 1).

### VENDORS RECEIVE PRESSURES FROM POLLUTED AIR AND NOISE

Workers reported experiencing environmental pressures on a daily basis due to high temperatures, climate changes, atmospheric pollution, and noise, acknowledging that “the environmental issue is really strong. Medellín is under construction and the air is really polluted” (MGFC); “our point of sale is exposed to the sun, to water...” (HGF6); “noise is frequent”. Our fellow workers themselves make a lot of fuss...” (MGFC), thus concluding that everybody contributes to environmental pollution in the downtown area (Figure 1).

### EFFECTS ARISING FROM PRESSURES RECEIVED AND GENERATED

Effects manifest in workers’ life and health conditions, because physical, mental and emotional health is vulnerable to pressures received and generated. Their fragile health status, in turn, affects their work: “when it rains the merchandise must be covered, we don’t sell, and this makes us feel psychologically unwell; and when the weather is too hot, we have problems like skin cancer. The truth is that climate affects health very much: noise, dust, that is, all contamination; working in the streets is freaking bad” (HGFG).

This group of workers reported being overwhelmed by formal trade and police, which see them as obstacles to market their products, because; “... then there is formal trade, informal trade, so coexistence is poor” (HGFG). Conversely, hostile relations with fellow workers and little solidarity impair their life and health conditions; “constant fights among vendors themselves, poor coexistence, this generates stress, not selling anything, and there are problems or quarrels among fellow workers...” (HGFG).

### FOR FEMALE STREET VENDORS, BOTH PRESSURES AND EFFECTS ARE GREATER

**Figure 2:**
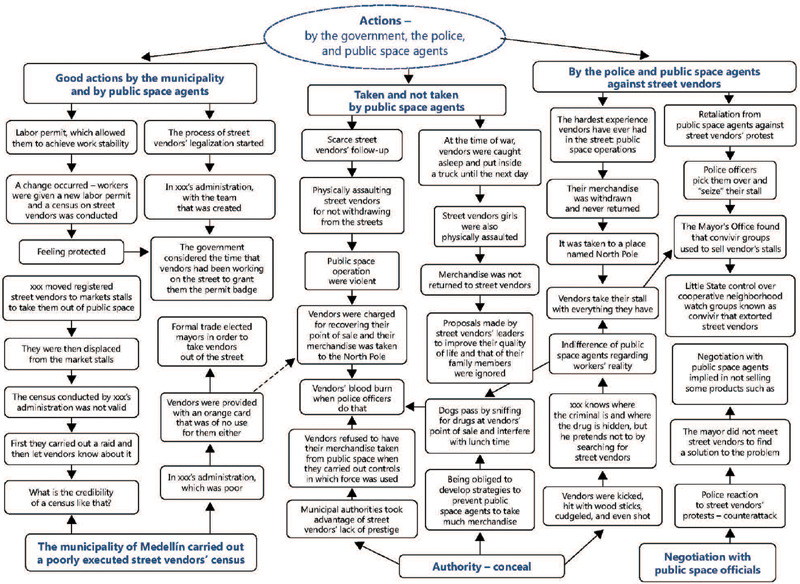
Actions of the government, the police, and public space agents on street vendors and their work.

Female workers participating in this study felt discriminate for being a woman, despite acknowledging that effort and exhaustion equally affect everybody, due to the physical and emotional conditions experienced by workers; “in reality, women in the streets are exposed to the worst physical, psychological, moral humiliation, we are very easy preys in the streets, there are much harassment on women in the streets” (EMVLD2) (Figure 2).

### ACTIONS ON PRESSURES RECEIVED AND GENERATED, AND ON WORKERS’ HEALTH

Palliative actions when workers lose their health Some actions are conducted by force of illness, such as when workers stay at home due to illness or are obliged to see a doctor because their health conditions can be deemed severe, as reported by the following statement: “I spent 37 days on intensive care” (MVM1).

#### Negative actions by the State

Street vendors experienced hostile actions and apathy towards situations that put them at risk, and recall that “public space operations were violent” (EMVLA1) and even questioned their honesty when, for example, conducting “control” actions against sale of drugs; sometimes these agents would assault street vendors, as reported next: “xxx, who walk around with some dogs, but they take screenshots to show to the cameras if they’re exerting authority, that they’re doing their job, they are searching for street vendors’ stalls” (HV1O) (Figure 2).

A municipal administration carried out the street vendors’ census, which, according to some State officials, was intended to make “street vendors feel protected” (MVLD1); a situation that could favor them or not, by attempting to relocate them, only from the State perspective, with the premise of providing them with better life conditions, because they would be exposed to the sun and to water. However, some participants in this study believe that the census had a negative connotation and was little reliable, because, “first they carried out a raid and then let vendors know about it; so, what’s the credibility of a census like that?” (HV2O), and for these reason street vendors believe that “the census was poorly executed” (HV2O). (Figure 2).

#### Positive actions from the State

Street vendors have also experienced positive actions to strengthen their activity as a profession and make their work in the streets easier; “in [xxx]’s administration, a new process started for us, it was when the change started. Because then [xxx] mayor, [xxx] Government’s Secretary, we started a process of dialogue, we proposed our demands to the mayor at that time when we were victims; and he designated the government’s secretary to deal directly with the issue of streets vendors” (HV1O). (Figure 2).

#### Workers’ actions against pressures received

Actions could eventually become violent, when they received aggressions from the State; “we had to reveal ourselves then, even my entire family saw me; because I’m a big fighter, defending one’s position, I’m very calm, but when a thing like this happens we must fight to defend ourselves at this time” (EMVA1). (Figure 2).

## DISCUSSION

Informal workers earn low income and have an unstable subsistence job as independent workers,^8^ and in Latin America, most of these workers occupy public space to develop their activities, which is why they are also lead players in changes of urban spaces^9^ and the territories they occupy.

Labor informality rates were higher than 50.0%, both in Latin America Latina and the Caribbean^10^ and in Colombia for 2020, and, according to estimates for the DANE accounted for 46.4% of employed workers.^4^ Most of these workers are men and women who turn the streets and sidewalks into their workplace, being known as “*venteros*” in Medellín, Colombia, These vendors work in subsistence jobs and provide their products at stalls installed directly on public roads or on sidewalks/platform, a situation that make them occupy a territory in an irregular and unsafe manner both for them and for pedestrians and other legal and illegal actors who develop their daily activities in downtown Medellin, as reported in other municipalities in the country.^11^

In relation to territory, it consists of a space and a place where social conflicts are generated, mediated by power relationships that turn into tension experiences difficult to understand, because it is where survival activities are developed,^12^ as evidenced in the present study, which found that workers should promote actions to respond to pressures received and generated in the public space and that affect their life, health, and working conditions, putting at risk their survival at that of the people for which they are responsible.^13^ These conditions are similar to those observed in informal workers in the streets of Bogotá, in the area of San Victorino,^14^ who, due to their labor condition, were marginalized from State protection, having difficulties in affiliating and accessing care services from the System of Social Security in Health and having no or scarce affiliation to the Pension System.

In Latin America and the Caribbean, some studies describe the factors that contribute to the increase in informal street vending. In Bolivia, men enter the informal economy due to lack of opportunities, and women because they need to help increase family income.^10^ In Peru, informal vendors account for 25.0% of the economically active population in mid-sized municipalities, and nearly 60.0% of these vendors are women.^10^ In Brazil street vendors set their own rules, such as flexible schedules; however, they have difficulties with the State and with regulations for the use of territory.^15^ Finally, in Argentina, labor informality is a phenomenon that affects 4 out of every 10 workers and is the main limited economic activity in the country.^15^

A study conducted in Medellin in 2014,^16^ in which 153 legalized street vendors were interviewed, reported that there should be an improvement in the conditions of the streets where these vendors work: restricted traffic of motor vehicles and provision of public lighting, green areas, and tree planting; standardized stalls; item for waste disposal and surveillance. The same study also observed that these actions would favor workers’ safety and provide them with greater comfort in the development of their activities.^16^

Another study whose objective was to describe the life and working conditions of informal street vendors in the roadway corridor named Ayacucho, in the city of Medellin, found that the cause of street vending in the city was displacement due to conflicts and lack of life opportunities.^17^ and that, once they are in the streets, they are affected by noise and visual pollution and by invasion of public space, which changes pedestrians’ mobility and blocks vehicle traffic,^17^ similar to what was evidenced in the present study.

In Cartagena, Colombia, a study conducted with informal vendors at Bazurto market observed that workers have an unfavorable work environment, being exposed to noise, pollution, and particles that affect their health conditions,^18^ conditions similar to those recorded in informal workers in Popayán, Colombia.^19^ These conditions were similar to those reported for street vendors in downtown, and can also affect their life and health conditions, with noise being the risk factor most identified by street vendors.^16^ It was also found that street vendors in downtown Medellín are exposed to ergonomic, psychosocial, safety, physical, chemical, and environmental risk factors that change and affect their health state.^16^

In relation to mental health, a study conducted with 258 street vendors in Chile, they reported being very happy with their families, but less happier regarding their friends; additionally, they reported being physically or mentally ill from 5 to 6 days per month.^20^ En Villavicencio, Colombia, with regard to health perception, informal vendors, it was observed that 45.4% had poor health, did not experience adequate living conditions, and were mentally exhausted.^21^ This previous evidence is coherent with the statements provided by the workers participating in the present study, who felt physically and mentally overwhelmed, ill, and exhausted due to their working conditions.

In Colombia, physical health problems were reported among informal workers, such as headache, fatigue, back pain, hearing loss, among others^16^; however, regarding life and working conditions of people who work as street vendors, there is still scarce comprehensive evidence on activities related to health promotion and prevention of diseases derived from exposure to heavy loads and incorrect postures and movements, which impair health and compromise physical capability and performance.^19^ It is also worth mentioning that these conditions coincide with those experienced by workers participating in the present study. However, a study that investigated the attitudes of street vendors in the Chapinero neighborhood in Bogotá,^22^ towards labor and political conditions found that the police is valued as an institution that provides safety, although it can be often absent. Vendors also reported that there is no management of policies to recovery public space; a finding similar to that observed for the workers participating in the present study, who reported that instability in safety.

It is important to bear in mind that the comprehensive evidence reported to compare the results of the present study is limited in some aspects, as occurs with topics related to the conflict generated with the municipal government, which conducted activities such as a street vendors’ census. Workers report that “the census was poorly executed,” since they considered that its data did not reflect reality, both regarding the number of workers and their life and working conditions.

With regard to illegal actors, drugs dealers, and those who lend money with usury interest rates (gota-gota illegal money lenders), there is no reported information, and this would be a new contribution to the lack of knowledge on factors that affect the health of street vendors, who experience stressful and pressure conditions, in their pursuit to cover their debts and interests they must pay to their lenders to have enough money and be able to work. The limitation of the present study was its difficulty to compare its results in several aspects explored, given the limited scientific evidence comprehensively generated to advance in understanding of the life and conditions of informal workers who perform their work activities in the streets and sidewalks, and this would be a line of research that should be further explored.

## CONCLUSIONS

Workers participating in this study enter street vending as a survival option, develop different labor activities to earn an income, experience stressful situations that become a barrier to achieve, maintain, or improve their life and health conditions.

In general, the health of these workers is affected by the conditions to which they are exposed during the development of their daily activities in a hostile environment in which workers generate and receive pressures, with diseases occurring due to exposure to external factors that increase their physical and mental weakness, making it difficult for them, in turn, to have better working conditions, in a space where struggle for use of public space by these workers results in worse life and health conditions.
